# Self-Assembly of a Triphenylene-Based Electron Donor Molecule on Graphene: Structural and Electronic Properties

**DOI:** 10.1021/acs.jpcc.1c10266

**Published:** 2022-06-01

**Authors:** Joris de la Rie, Mihaela Enache, Qiankun Wang, Wenbo Lu, Milan Kivala, Meike Stöhr

**Affiliations:** † Zernike Institute for Advanced Materials, University of Groningen, Nijenborgh 4, Groningen 9747 AG, The Netherlands; ‡ Institute of Organic Chemistry, University of Heidelberg, Im Neuenheimer Feld 270, Heidelberg 69120, Germany; § Centre for Advanced Materials, University of Heidelberg, Im Neuenheimer Feld 225, Heidelberg 69120, Germany

## Abstract

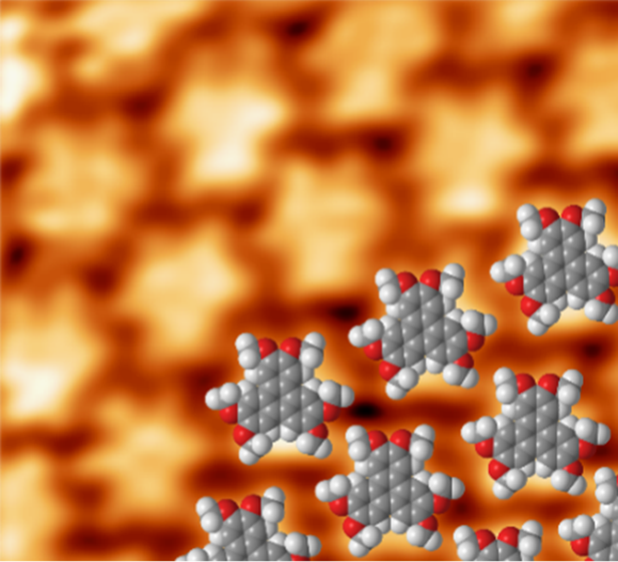

In this study, we report on the self-assembly of the organic electron donor 2,3,6,7,10,11-hexamethoxytriphenylene (HAT) on graphene grown epitaxially on Ir(111). Using scanning tunneling microscopy and low-energy electron diffraction, we find that a monolayer of HAT assembles in a commensurate close-packed hexagonal network on graphene/Ir(111). X-ray and ultraviolet photoelectron spectroscopy measurements indicate that no charge transfer between the HAT molecules and the graphene/Ir(111) substrate takes place, while the work function decreases slightly. This demonstrates that the HAT/graphene interface is weakly interacting. The fact that the molecules nonetheless form a commensurate network deviates from what is established for adsorption of organic molecules on metallic substrates where commensurate overlayers are mainly observed for strongly interacting systems.

## Introduction

Since its first successful isolation, graphene^1^ has developed into one of the most promising materials for many electronic applications due to, among others, its excellent electrical, thermal, and mechanical properties.^2,3^ For integration of graphene into devices such as light-emitting diodes,^4^ field-effect transistors,^5^ batteries,^6^ solar cells,^7^ or as an electrode,^8,9^ it is important to align graphene’s electronic levels with other materials to achieve optimal device performance.

Depending on the substrate it is grown on, a varying charge carrier concentration and work function can be obtained.^10−12^ However, as often a finer control over these properties is desired, many methods have so far been explored to further modify graphene’s electronic properties. Gating allows rapid control over both charge carrier concentration and type but suffers from large parasitic capacitance.^13^ Chemical covalent functionalization, with, for example, nitrogen or hydrogen, offers a scalable path to controlled doping, but the chemical bonds formed this way often break the sp^2^ hybridization of the graphene lattice, which drastically lowers charge carrier mobility.^14^ Noncovalent adsorption of small molecules (e.g., H_2_O, O_2_, and NH_3_) avoids this problem but is highly sensitive to changes in the environment.^15^ Adsorption of metal atoms^16^ or nanoparticles^17^ should be a more robust approach, but the most promising results also show signs of chemisorption, which would bring the same problem as covalent functionalization.^18^

Recently, noncovalent functionalization of graphene by adsorption of planar pi-conjugated organic molecules has attracted increasing attention^15,19−21^ since these molecules noncovalently interact with graphene through π–π interactions which do not perturb the graphene sp^2^ lattice, leaving its high charge carrier mobility intact. It has been shown, both on metal surfaces^22^ and on graphene,^23,24^ that the orientation of the molecules—both the in-plane and the out-of-plane orientation—in the first layer on the surface strongly affects the intermolecular and molecule–surface interactions and thereby the level of doping and change in work function. The interaction strength at the graphene/metal interface also plays a role as molecules tend to bind more strongly to graphene if the graphene–substrate interactions are stronger.^25,26^ Of the recent studies on this topic, many have focused on p-type doping of graphene by adsorption of the organic acceptor tetracyano-*p*-quinodimethane (TCNQ), whereas a variety of substrates has been used for graphene.^24,27−29^ The degree of doping can be controlled by the coverage, the replacement of TCNQ by its derivatives,^24,25^ or a combination with molecular donors to form charge-transfer complexes.^21,30^ The so far most commonly employed donor molecules are (metallo-)phthalocyanines^28,31^ and tetrathiafulvalene.^21,30,32^ This leaves the class of triphenylene derivatives underexplored. Triphenylene can be easily functionalized and thus can act as both acceptor or donor molecules.^33−36^ To the best of our knowledge, only the acceptor hexacyano-hexaazatriphenylene has been studied on graphene to date.^23,37^

**Scheme 1:**
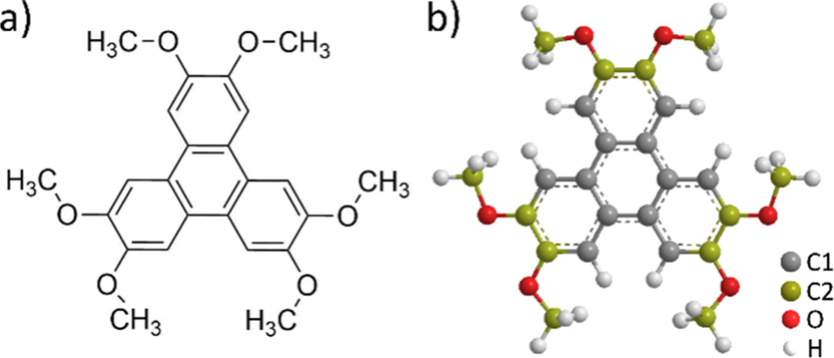
Chemical Structure of HAT: (a) Chemical Structure of HAT; (b) Ball and Stick Model Indicating the Species of Carbon Atoms Distinguished in the XPS Analysis, Which are Labeled in Gray and Yellow.

In this contribution, we investigate the behavior of the triphenylene-derived donor molecule 2,3,6,7,10,11-hexamethoxytriphenylene (HAT, see Scheme 1). HAT has been used as an organic donor in combination with a variety of organic acceptors in charge-transfer crystals^38−40^ and charge-transfer complexes on metal surfaces.^41^ Here, we use scanning tunneling microscopy (STM), low-energy electron diffraction (LEED), and ultraviolet/X-ray photoelectron spectroscopy (UPS/XPS) to study its structural and electronic properties on slightly p-type doped graphene grown on Ir(111).^42^

## Experimental Section

### Synthesis

HAT was synthesized in accordance with literature procedures.^34,36,43^

### Sample Preparation

The graphene on the Ir(111) substrate was prepared in an ultrahigh vacuum (UHV) system (base pressure below 10^–10^ mbar). Clean Ir(111) crystals were prepared by cycles of argon ion sputtering and annealing at 1300 K. Single-layer graphene was grown by chemical vapor deposition by exposing the Ir(111) substrate to a partial pressure of 4 × 10^–7^ mbar of ethylene (C_2_H_4_) gas for 4 min while the substrate was held at 1200 K. The graphene/Ir(111) samples were then transferred through ambient air to the preparation chamber (base pressure below 2 × 10^–9^ mbar) of a two-chamber UHV system. After transfer, the graphene/Ir(111) samples were annealed at 750 K for 60 min to ensure clean surfaces. The HAT molecules were sublimed using a commercially available Knudsen cell evaporator (CreaPhys) with the deposition rate monitored by a water-cooled quartz crystal microbalance (QMB). The sample was kept at room temperature during deposition.

### Measurements

Most measurements were carried out in the analysis chamber (base pressure below 10^–9^ mbar) of the aforementioned two-chamber UHV system, with the samples held at room temperature. The analysis chamber houses a variable temperature scanning tunneling microscope (Scienta Omicron GmbH, operated at room temperature), LEED optics (SPECS), a hemispherical analyzer (PREVAC EA15), a twin anode X-ray source, and a He discharge lamp. The STM measurements were carried out in the constant current mode with a mechanically cut Pt/Ir tip. Bias voltages are reported with respect to a grounded sample. The STM data were processed using WSxM software.^44^ LEED patterns were simulated using LEEDpat.^45^ UPS measurements were performed using He I radiation (21.2 eV) at an emission angle of 40° with respect to surface normal (valence band) and 0° (secondary electron cutoff). XPS measurements were performed using Mg Kα radiation (1253.6 eV) at normal emission. A few measurements were performed at the same UHV system at which the graphene preparation was carried out. These measurements were performed using a low-temperature STM instrument (Scienta Omicron GmbH, operated at 77 K) and a microchannel plate LEED (Scienta Omicron GmbH).

## Results and Discussion

**Figure 1:**
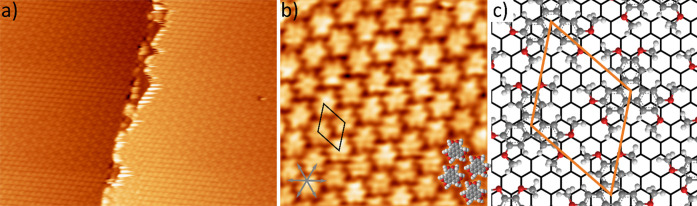
Self-assembly of HAT on graphene on Ir(111). (a) Overview STM image (66 × 49 nm^2^, *U* = 2.1 V, *I* = 10 pA, and *T* = 77 K) in which both the HAT molecules and the graphene/Ir(111) Moiré pattern are resolved on both sides of a step edge of the Ir(111) substrate. (b) STM image (10 × 10 nm^2^, *U* = −1.8 V, *I* = 10 pA, and *T* = 300 K) of the close-packed network for sub-monolayer coverage. The unit cell is indicated by a black rhombus, and some molecules are superimposed to indicate their arrangement. (c) Tentative structure model for HAT on graphene on Ir(111). The unit cell is indicated in orange, and the graphene lattice is indicated in black.

**Figure 2:**
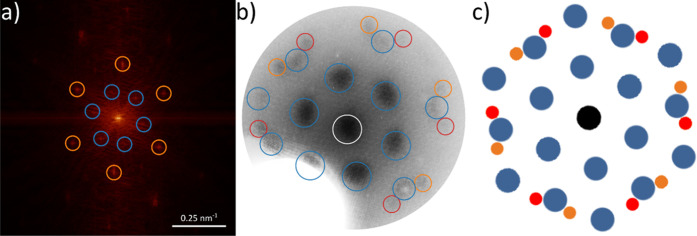
Reciprocal space data of the HAT network on graphene/Ir(111). (a) FFT of the STM image shown in Figure 1a (scale bar in white). The spots from the Moiré pattern are marked in blue, and those from the HAT network are marked in orange. (b) LEED pattern acquired at 28.8 eV. The (00) spot is marked in white, the Moiré spots are marked in blue, and the HAT spots are marked in orange and red. (c) Simulated LEED pattern. More details on (a,b) can be found in Supporting Information, Figures S2 and S6.

For a molecular coverage up to one monolayer of HAT on graphene/Ir(111), the molecules assembled in a hexagonal close-packed network, as can be seen in the STM image in Figure 1a. Individual molecules can be easily identified in Figure 1b by their characteristic shape, exhibiting six protrusions at the periphery. The hexagonal network exhibited a long-range order extending several hundreds of nanometers (for an overview image showing the long-range order, see Figure 1a; an STM image of a larger area is presented in the Supporting Information in Figure S1). The tentative structure model is shown in Figure 1c. This network is stabilized by hydrogen bonds between the oxygen atoms in one molecule and the hydrogen atoms in the neighboring molecule’s methyl group. The unit cell of the hexagonal network is indicated by an orange rhombus and has a size of 1.30 nm × 1.30 nm ± 0.05 nm with an enclosing angle of 60°. The structural relationship between the HAT network and the graphene substrate is established by combining the information obtained from fast Fourier transformation (FFT) of the STM data and the LEED data (Figures 2 and S3–S7 in the Supporting Information). The FFT confirms the size of the unit cell (see Figure S2 in the Supporting Information), and both the FFT and LEED data agree exceptionally well with the simulated LEED pattern. The network, a commensurate  superstructure, exhibits mirror symmetry, and the domains enclose an angle of ±19 ± 1° with the principal directions of graphene. From the commensurate arrangement of the molecules, we conclude that the graphene HAT interaction is responsible for this, and we will discuss this further below. It should be noted that the self-assembly is not influenced by the graphene/Ir(111) Moiré pattern.

The molecular arrangement and unit cell are very similar to the ones found for HAT on Ag(111), where the molecules were also found to assemble in a hexagonal packing with a lattice parameter of 1.32 nm.^41^

**Figure 3:**
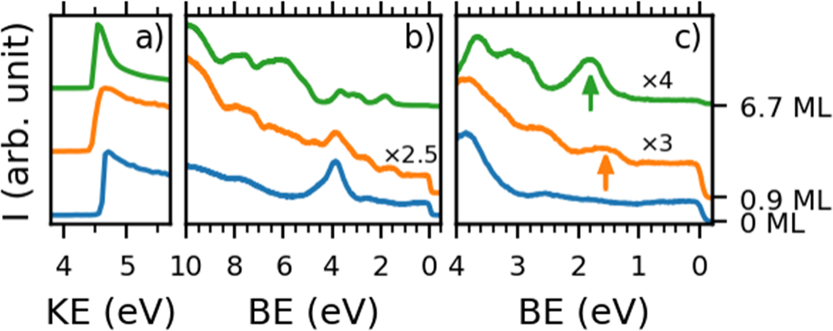
UPS spectra for pristine graphene on Ir(111) (blue, 0 ML), a coverage close to a monolayer of HAT (orange, 0.9 ML), and a multilayer of HAT (green, 6.7 ML) on graphene/Ir(111), measured at an emission angle of 40°. (a) Secondary electron cutoff region taken with a bias of −5.0 V applied to the sample for determining the work function. (b) Overview UPS spectrum. (c) Close-up UPS spectrum to better visualize the features around the Fermi level. Arrows indicate the position of the HAT HOMO.

To gain insights into the electronic properties and molecule–substrate interactions, we performed photoelectron spectroscopy measurements. The UPS data are displayed in Figure 3. The valence band spectra were acquired at an emission angle of 40° to prevent overlap of the signal originating from the Ir(111) surface state and the HAT highest occupied molecular orbital (HOMO), which is significant at 0° (spectra acquired at an emission angle of 0° can be found in the Supporting Information, see Figures S8 and S9).

For a coverage of close to a monolayer (0.9 ML in Figure 3), we can identify the HOMO level at 1.5 eV, and for the multilayer sample, we can identify the HOMO level at 1.8 eV. Similar shifts are also observed for the higher HOMO levels. This can be attributed to final-state hole screening by the metal substrate, which is the strongest for the first molecular layer.^46,47^ No additional features (from, e.g., a hybrid state or former lowest unoccupied molecular orbital) are observed, and the relative positions of the HOMO levels agree well with those of HAT on Ag(111), a weakly interacting interface^41^ (for UPS data of HAT on Ag(111), see Figure S10). From this, we can conclude that HAT also interacts weakly with graphene, which can be most likely classified as physisorption.

With UPS measurements, we also determined the work function of our samples by applying a bias of −5 V to the sample and recording the secondary electron cutoff region (left panel in Figure 3). For pristine graphene on Ir(111), we found a work function of Φ = 4.6 eV in good agreement with literature values.^48^ Upon deposition of a monolayer of HAT, the work function decreased to Φ = 4.4 eV and remained at this value as further layers were deposited.

**Figure 4:**
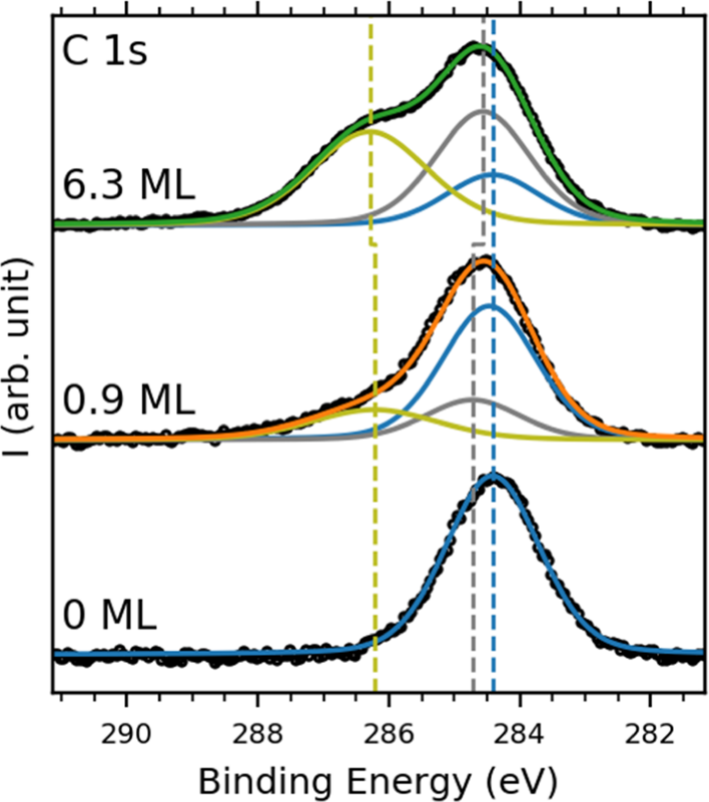
C 1s XPS spectra of pristine graphene on Ir(111) (blue), close to monolayer coverage of HAT (orange) and multilayer coverage of HAT (green) on graphene on Ir(111). The black circles indicate the measured data, and the solid lines indicate the fit envelopes. The individual fits are indicated in blue (graphene), gray (HAT C1), and yellow (HAT C2), with the dashed lines indicating the peak positions.

**Figure 5:**
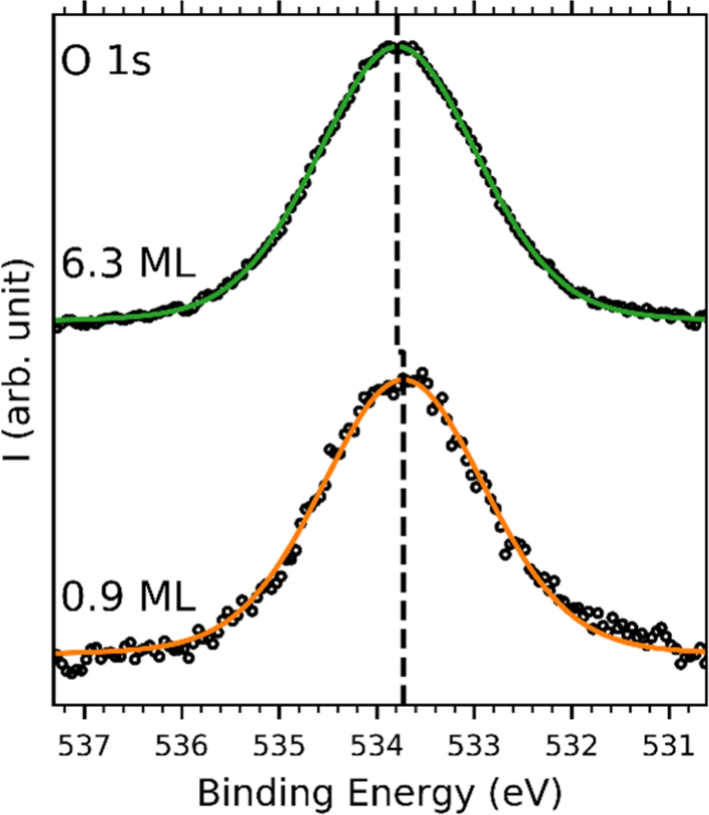
O 1s XPS spectra of close to monolayer coverage of HAT (orange) and multilayer coverage of HAT (green) on graphene on Ir(111). The black circles indicate the measured data, and the solid lines indicate the fit envelopes. The peak position is indicated by the black dashed line.

We also obtained XPS spectra for the C 1s (Figure 4) and O 1s (Figure 5) core levels for varying coverage levels of HAT on graphene on Ir(111). The C 1s spectrum for graphene/Ir(111) shows a single peak centered at 284.4 eV. We use this peak’s position and full width at half-maximum (fwhm) to represent graphene in the mono- and multilayer HAT spectra. For fitting the C 1s spectra obtained for close to monolayer and multilayer coverage of HAT, we added two additional peaks originating from the HAT C1 atoms (gray, the twelve carbon atoms not bonded to oxygen in the triphenylene backbone, see Scheme 1b) and HAT C2 (yellow, the twelve carbon atoms bonded to oxygen, see Scheme 1b). As these two species are equally abundant in the HAT molecule, we used a 1:1 area ratio when fitting these peaks. The graphene/HAT ratio for the C 1s spectrum was determined by accounting for attenuation from the HAT layer(s), with the HAT thickness determined by the QMB during deposition (see the Supporting Information for further details). We found these peaks at 284.7 and 286.2 eV, respectively, in line with previously reported values for HAT adsorbed on Ag(111)^41^ and SiO_2_.^49^ The O 1s spectra were fitted using a single peak at 533.7 eV. This value is in good agreement with earlier results when using Ag(111) as the substrate^41^ but 0.7 eV higher than reported for HAT on SiO_2_.^49^ This difference might originate from the different energy level alignments of HAT on these different substrates. More details on the peak parameters can be found in Table 1.

**Table 1:** Fitting Parameters for XPS Data^a^

	C 1s			O 1s
	graphene	HAT C1	HAT C2	HAT O
Gr/Ir(111)	284.4/1.7			
	100%			
monolayer	284.4/1.7	284.7/1.7	286.2/2.3	533.7/2.0
HAT	62%	19%	19%	100%
multilayer	284.4/1.7	284.6/1.7	286.3/2.1	533.8/2.0
HAT	18%	41%	41%	100%

a XPS binding energies in eV/fwhm in eV and relative area (%) for the C 1s and O 1s core levels.

As the HAT coverage increased from the monolayer to multilayer, no significant binding energy shifts were observed for the C 1s or O 1s peak positions within our resolution of ±0.1 eV. Note that a core-hole screening effect similar to the UPS data cannot be observed due to the higher information depth of XPS, that is, the multilayer spectrum also obtains information from the molecule substrate interface and larger line width of the X-ray source compared to the UV source.

From our UPS and XPS data, we conclude that no charge transfer occurred between the HAT molecules and the graphene/Ir(111) substrate. A possible charge transfer could be identified by a shift of the peak positions between mono- and multilayer coverage, and the value of such shifts should be on the order of hundreds of meVs and apply to both C 1s and O 1s peak positions.^23,32,50−52^ In our data, the peaks originating from the HAT molecules appeared at the same energy for both mono- and multilayer coverage. Moreover, there is also no significant doping of the graphene present as this would induce a shift in the graphene C 1s peak.^25^ In particular, additional peaks in the vicinity of the HOMO level would indicate charge transfer; the absence of such features in our data therefore indicates that no charge transfer takes place.^53,54^

**Figure 6:**
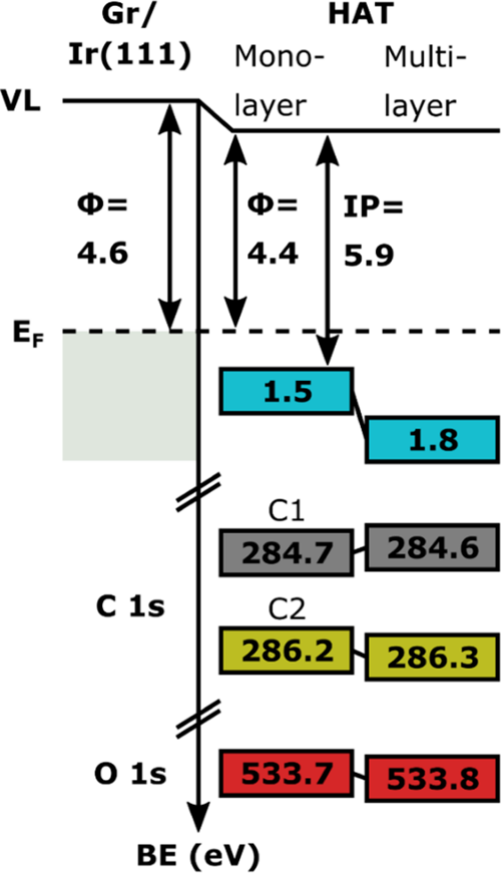
Diagram displaying the energy level alignment of HAT on graphene on Ir(111). Shown are the work function (φ), ionization potential (IP), HOMO (cyan), and C 1s and O 1s core levels for both mono- and multilayer of HAT (for the color coding, see Scheme 1b).

We then finally turn to the decrease in the work function of graphene/Ir(111) upon adsorption of a monolayer of HAT. We attribute this decrease to the Pauli repulsion (also known as the *push-back* or *pillow effect*) between the electrons of the HAT molecule and the electron cloud of the substrate extending into the vacuum, well known for molecules adsorbed on metal surfaces.^55^ This effect has also been predicted for molecule/graphene/metal systems but tends to be overshadowed by charge-transfer-induced dipoles.^25^ As no charge transfer occurs in the HAT/graphene/Ir(111) system, the measured work function shift is due to only the push-back effect. The energy level alignment of HAT on graphene on Ir(111) is summarized in Figure 6.

Our photoelectron spectroscopy data suggest that there is little interaction between the HAT molecules and the graphene on the Ir(111) surface. This is not too unusual, as epitaxial graphene has been often—although not always^25^ reported to passivate metal–organic interfaces.^56−60^ Nonetheless, the HAT monolayer appears to be commensurate with the graphene substrate. This is in contrast to weakly interacting metal–organic systems for which normally incommensurate molecular overlayer structures have been reported,^61−64^ while commensurate overlayer structures are (almost) only formed when the molecule substrate interactions exceed the intermolecular interactions, that is, the molecules want to interact in clearly preferred ways with the substrate.^52,65,66^ Possibly, it is a coincidence that the size of the molecular unit cell is very close to a multiple of the graphene unit cell. However, if that was the case, we should observe many rotational domains. Instead, we observe just one domain (and its equivalent mirror domain). Even if the structure is not exactly commensurate, it still forms a point-on-line coincidence.^67,68^ This speaks for a molecule–substrate interaction that is optimized by the HAT on graphene on the Ir(111) system, resulting in a single orientation. Given that the molecular lattice appears to be commensurate with the graphene lattice (and not with the Ir(111) support or the Moiré pattern), it seems likely that this interaction occurs between the HAT molecules and the graphene layer. Possibly, π–π interactions between graphene and the aromatic backbone of HAT are responsible.^69^

## Conclusions

We studied the structural and electronic properties of an organic electron donor on graphene on Ir(111) using STM, LEED, UPS, and XPS. At sub-monolayer coverages, the molecules assembled in an, apparently commensurate, close-packed hexagonal structure. The two domains we observed are mirror images of each other with the [1–10] direction of graphene as the mirror direction. From the photoelectron spectroscopy measurements, we find neither evidence for charge transfer between HAT and graphene nor HAT and Ir(111), all together indicating a weak interaction (physisorption) between the HAT molecules and the graphene on the Ir(111) substrate. The fact that we nonetheless have clear indications for a commensurate molecular overlayer is in contrast to the established knowledge for metal–organic interfaces. This shows that even at weakly interacting interfaces, the substrate can play a significant role in guiding self-assembled structures.

## Supporting Information Available

Additional STM, LEED, and UPS (normal emission) data for HAT/graphene/Ir(111) and UPS data for HAT/Ag(111) (PDF)
