# Supplementary material for: Self-Assembly of a Triphenylene-Based Electron Donor Molecule on Graphene: Structural and Electronic Properties
Source: J Phys Chem C Nanomater Interfaces. 2022 Jun 1;126(23):9855–61. doi: 10.1021/acs.jpcc.1c10266 (PMC9207905; doi:10.1021/acs.jpcc.1c10266)
Supplement: Supplementary file 1 — jp1c10266_si_001.pdf [file jp1c10266_si_001.pdf]

Supporting Information (SI)

Self-assembly of a Triphenylene-Based Electron Donor Molecule on Graphene: Structural and Electronic Properties

Joris de la Rie[a], Mihaela Enache[a], Qiankun Wang[a], Wenbo Lu[a], Milan Kivala[b,c], Meike Stöhr*[a]*

[a] Zernike Institute for Advanced Materials, University of Groningen, Nijenborgh 4, 9747 AG Groningen (The Netherlands)

[b] Institute of Organic Chemistry, University of Heidelberg, Im Neuenheimer Feld 270, 69120 Heidelberg (Germany)

[c] Centre for Advanced Materials, University of Heidelberg, Im Neuenheimer Feld 225, 69120 Heidelberg (Germany)

Additional STM, LEED, UPS (normal emission, difference spectrum) and XPS (fitting information, stoichiometry) data for HAT/graphene/Ir(111) and UPS data for HAT/Ag(111).

Additional STM and LEED data for HAT/graphene/Ir(111)

An overview STM image for HAT on graphene on Ir(111) is shown in Figure S1, demonstrating the long-range order of the self-assembled network. Figure S2 shows an STM image and its fast Fourier transformation (FFT) for HAT on graphene on Ir(111), where both the molecules and the graphene Moiré pattern are resolved. Figure 3 shows an experimental (3a) and simulated (3b) LEED pattern of graphene on Ir(111). Figures S4, S5, S6 and S7 display the LEED pattern for a monolayer of HAT on graphene on Ir(111), focussed on the (00) spot (S4), extended to include the 1st order Ir(111) spots (S5), and at intermediate energies (S6, S7), respectively.

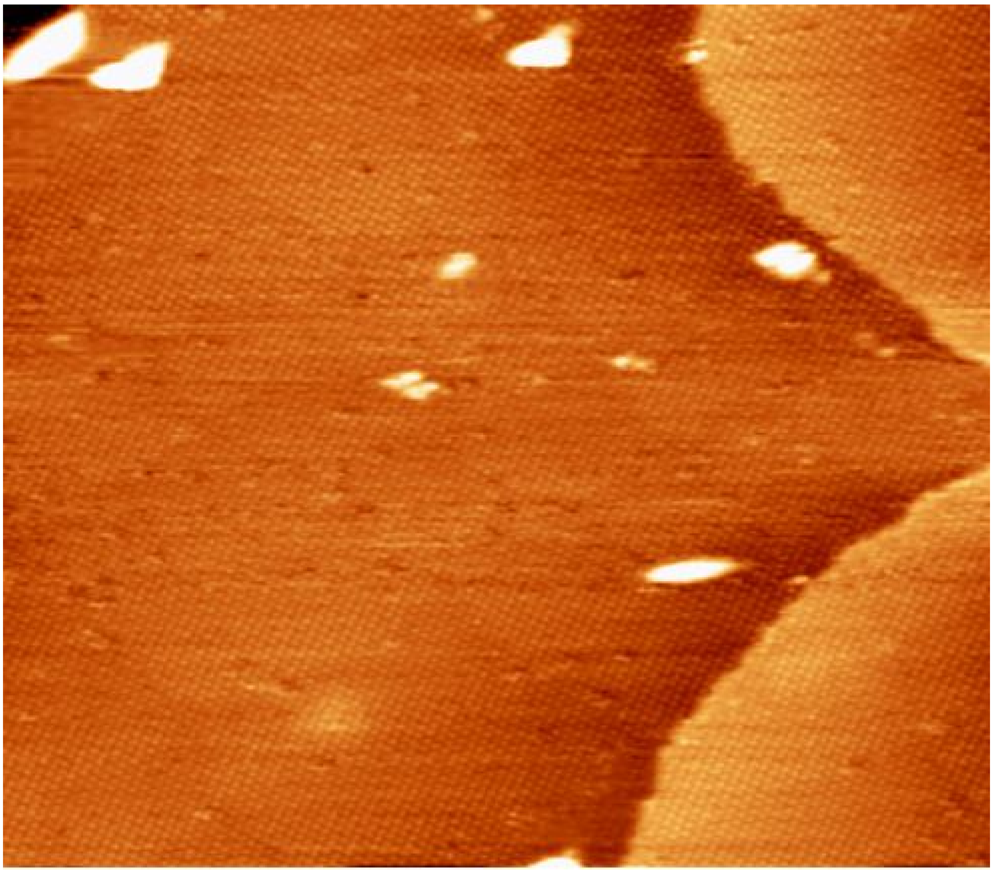

Figure S1: Overview STM image (100 x 87 nm^2, U = -1.8 V, I = 20 pA, T =300 K) of a monolayer of HAT on graphene on Ir(111). The step edges on the right side correspond to step edges of the Ir(111) substrate.

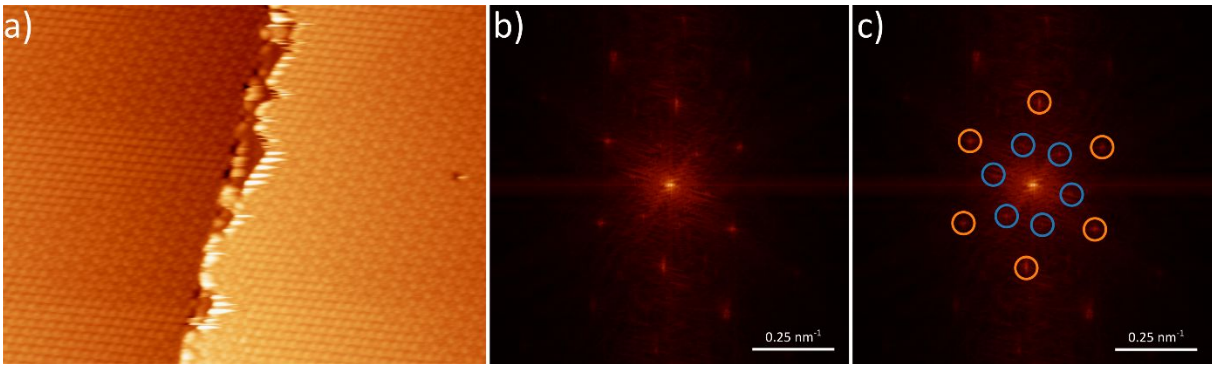

Figure S2: STM data with both the molecular lattice and the graphene/Ir(111) Moiré pattern resolved. a) STM image of HAT domains on both sides of an Ir(111) step edge. Both the molecular lattice and the Moiré pattern are resolved. Imaging parameters: 66 nm x 49 nm, U = 2.1 V, I = 10 pA, T = 77 K. b) Fast Fourier Transform (FFT) of the STM image in a) (scalebar in white). c) The same image as b). The circles mark the reciprocal lattice spots of the graphene Moiré pattern (blue) and HAT molecules (orange). The ratio between molecule:Moiré distances in the FFT is 1.95, which matches the expected ratio for a HAT lattice periodicity of 1.30 nm and a graphene/Ir(111) Moiré periodicity of 2.53 nm[1].

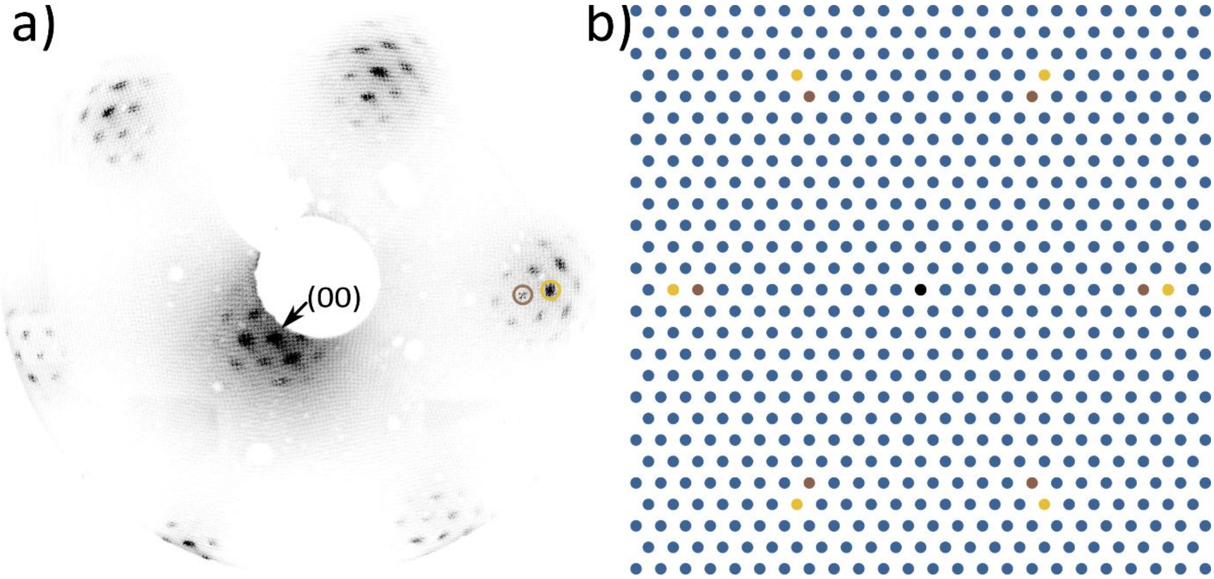

Figure S3: LEED pattern of graphene on Ir(111). a) Experimentally obtained pattern at 76 eV. The (00) spot is indicated by a black arrow. On the right hand side, the spots originating from the Ir(111) surface (brown circle) and graphene (yellow circle) are indicated. Further spots are due to the graphene/Ir(111) Moiré pattern. b) Simulated pattern. The black spot in the centre is the (00) spot, the brown and yellow spots are due to the Ir(111) and graphene, and the blue spots are due to the Moiré pattern.

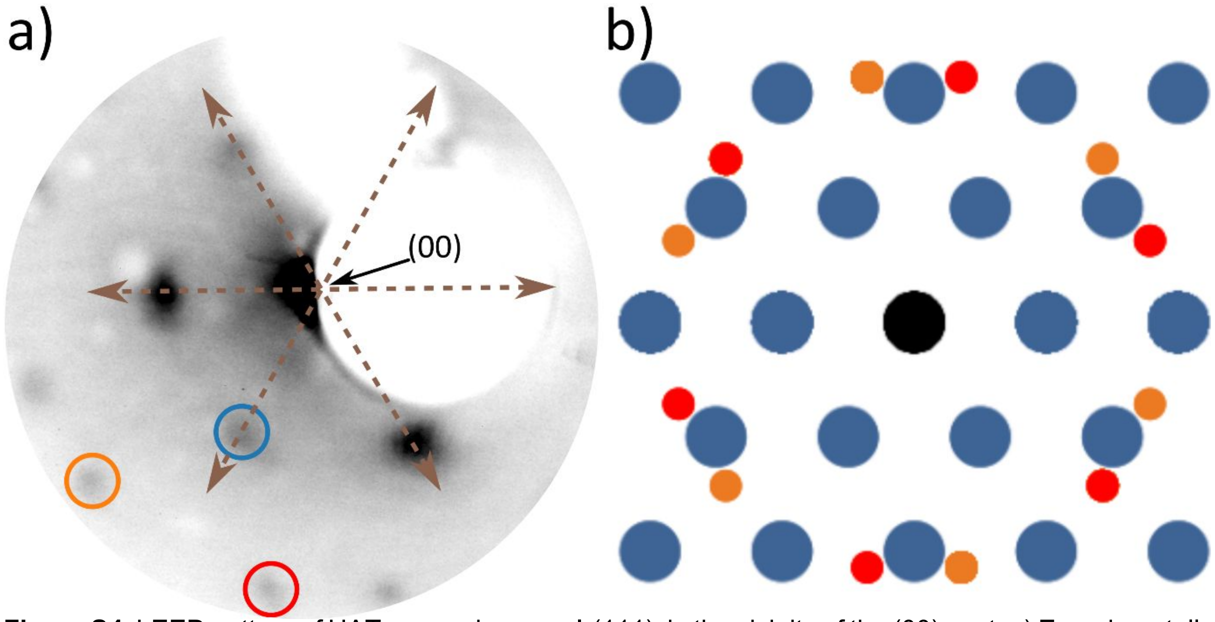

Figure S4. LEED pattern of HAT on graphene on Ir(111), in the vicinity of the (00) spot. a) Experimentally obtained LEED pattern for a monolayer of HAT on graphene/Ir(111), taken at a beam energy of 15.3 eV. The black arrow indicates the (00) spot, the blue circle highlights a 1st order graphene/Ir(111) Moiré spot and the orange and red circles highlight a 1st order spot from each mirror domain of the HAT assembly. The dashed brown arrows indicate the principal Ir(111) directions. b) Simulated LEED pattern for HAT on graphene/Ir(111), including the (00) spot (black), the graphene/Ir(111) Moiré spots (blue, 1st and 2nd order) and HAT molecule spots (orange and red, 1st order). Note that while our simulation and the LEED pattern for pristine graphene (Figure S2) include the 2nd order graphene/Ir(111) Moiré spots, in the experimental pattern in a) these are not distinguishable which is common for molecule/graphene systems: the outermost Moiré spots often are no longer visible after deposition of a layer of molecules [2, 3].

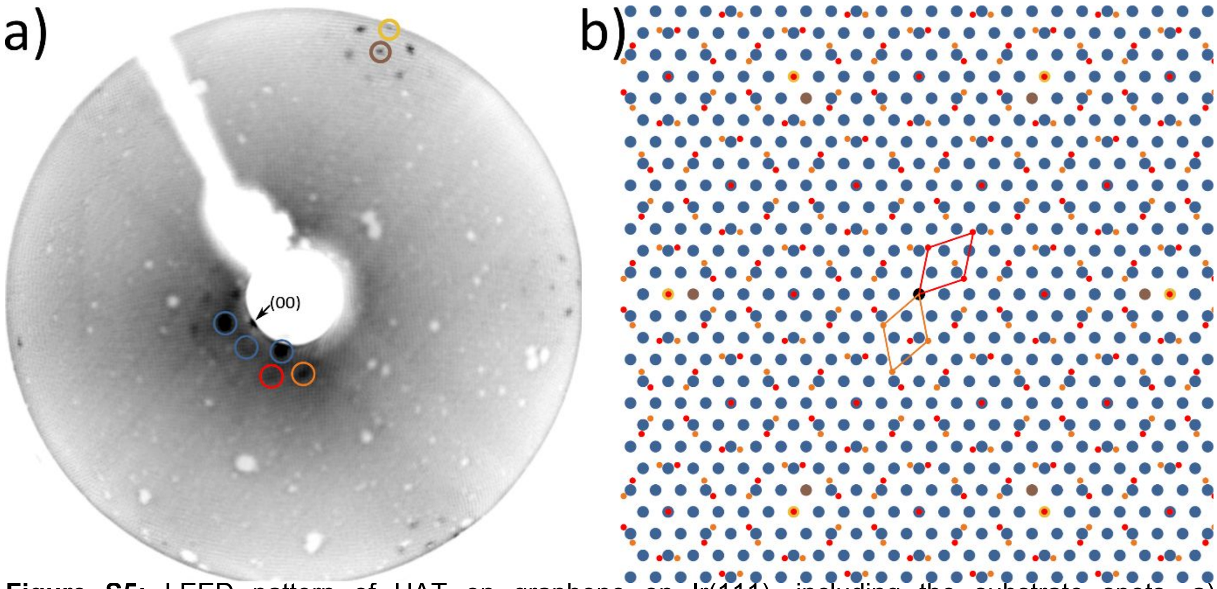

Figure S5: LEED pattern of HAT on graphene on Ir(111), including the substrate spots. a) Experimentally obtained LEED pattern of HAT on graphene on Ir(111), including the Ir(111) and graphene substrate spots, taken at a beam energy of 75.6 eV. The black arrow indicates the (00) spot, the blue circles highlight a few 1st order graphene/Ir(111) Moiré spots, the orange and red circles highlight a 1st order spot from each of the mirror domains of the HAT assembly, respectively. At the top of the image the brown circle highlights a Ir(111) spot and the yellow circle a graphene spot. The satellites around the substrate spots are due to the graphene/Ir(111) Moiré pattern. b) Simulated pattern. The black spot in the centre is the (00) spot, the brown and yellow spots are due to the Ir(111) and graphene, the blue spots are due to the Moiré pattern and the orange and red spots are due to the mirror domains of the HAT assembly. The orange and red diamonds indicate the unit cell for each of the mirror domains.

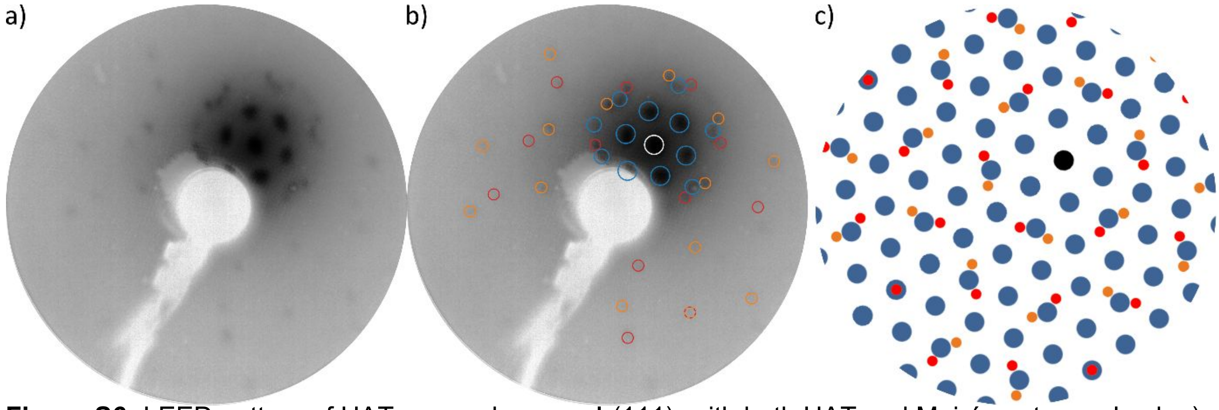

Figure S6: LEED pattern of HAT on graphene on Ir(111), with both HAT and Moiré spots resolved. a) Experimental LEED pattern obtained at 28.8 eV at the MCP-LEED. b) The same LEED pattern as in b). The (00) spot (white circle) is surrounded by 2 rings of Moiré pattern spots (blue). Directly outside the 2nd Moiré ring are the HAT spots (red and orange). Additionally, a variety of spots from higher order HAT diffractions are visible. c) Simulated LEED pattern matching the experimental pattern in a).

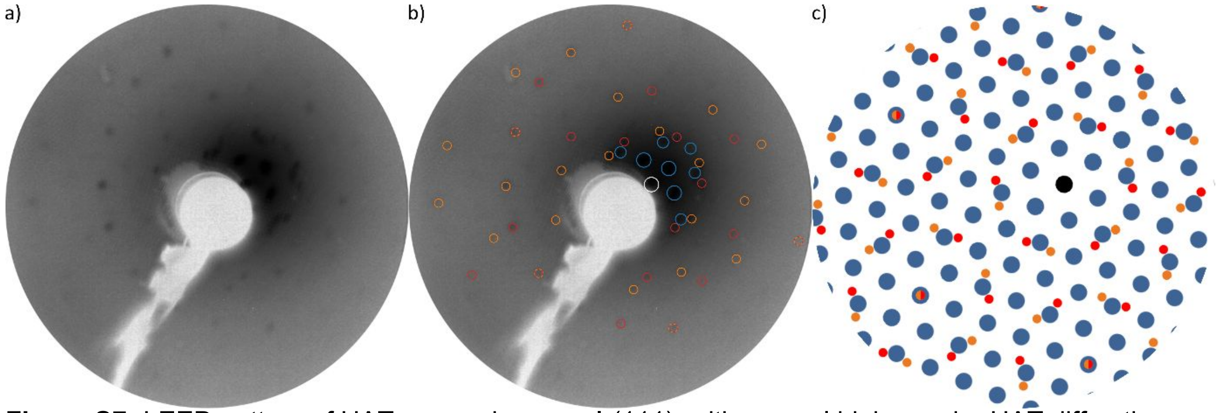

Figure S7: LEED pattern of HAT on graphene on Ir(111), with several higher-order HAT diffraction spots resolved. a) Experimental LEED pattern obtained at 40.6 eV at the MCP-LEED. b) The same pattern as in b). The (00) spot is marked in white and the Moiré pattern spots in blue. Spots due to the two HAT mirror domains are marked in red and orange, respectively. Four of these spots are formed by spots from both domains and thus, overlap (marked with a red-orange striped circle). c) Simulated LEED pattern. Overlapping spots from the two HAT domains are marked by a half red, half orange circle.

Additional ultraviolet photoelectron spectroscopy (UPS) data for HAT/graphene/Ir(111) and HAT/Ag(111)

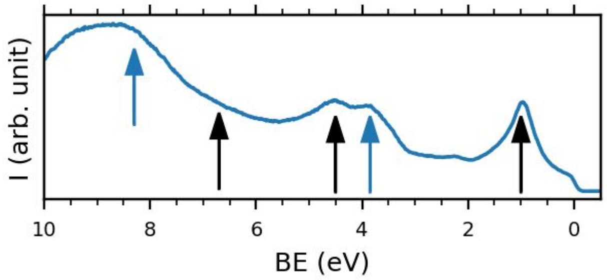

Figure S8: UPS spectrum for pristine graphene on Ir(111). Emission features from the Ir(111) bands are indicated by black arrows at 1.0, 4.5 and 6.7 eV. The graphene σ and π features are indicated by blue arrows at 3.8 and 8.2 eV, respectively. Spectrum acquired at normal emission.

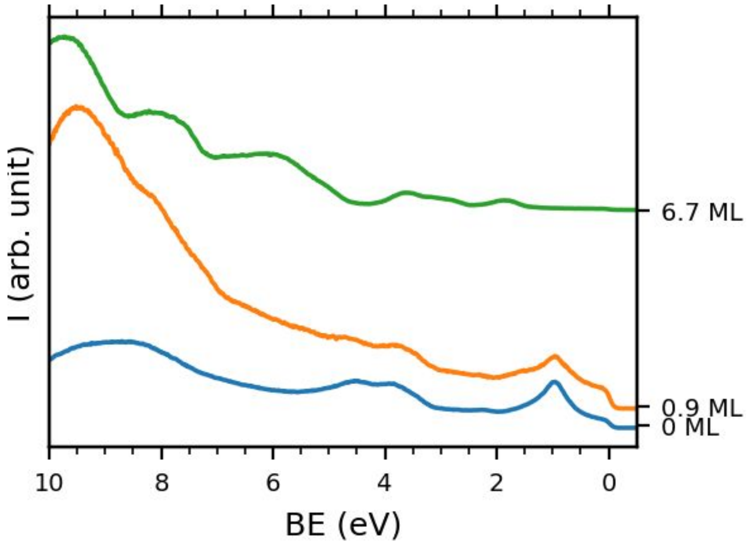

Figure S9: UPS spectra for pristine graphene on Ir(111) (blue), a close to monolayer coverage of HAT (orange) and a multilayer coverage of HAT (green) on graphene on Ir(111). Spectra acquired at normal emission.

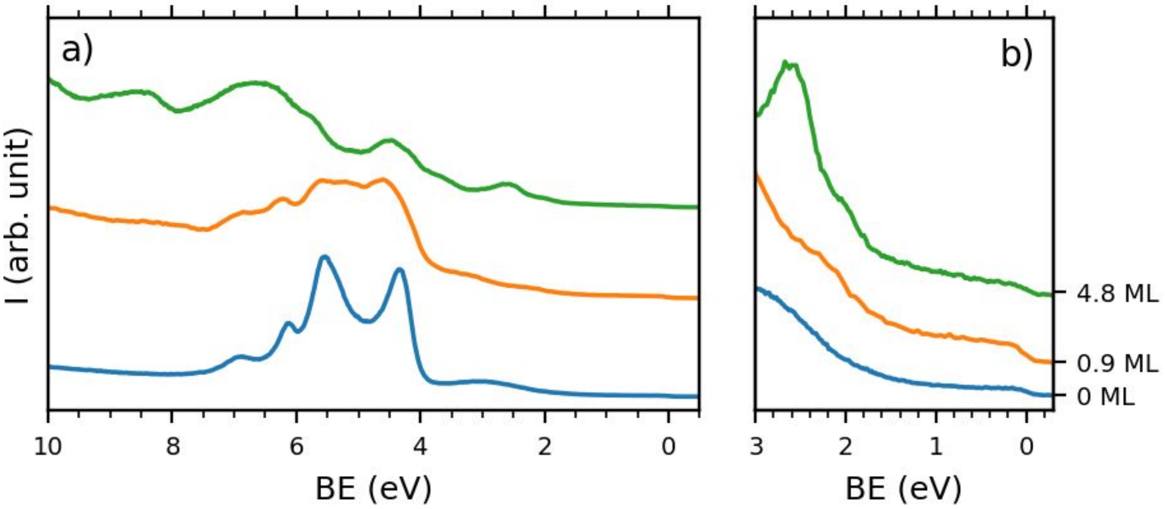

Figure S10: UPS spectra for Ag(111) (blue), a monolayer of HAT (orange) and a multilayer of HAT (green) on Ag(111), a) Survey spectrum and b) close-up of the HOMO level. Spectra acquired at normal emission. Note that the multilayer sample in this case is relatively thin and therefore the spectrum includes signal from the first layer of molecules, as can be seen most clearly in the monolayer HOMO at 2.2 eV, which appears as a shoulder of the HOMO of the succeeding layers.

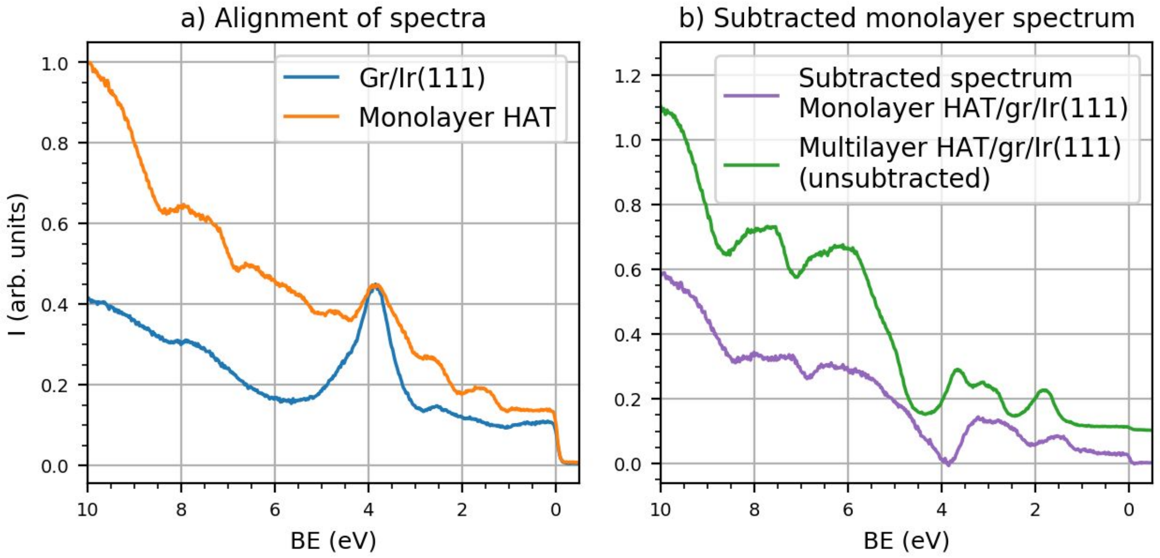

Figure S11: Difference spectrum for monolayer HAT/gr/Ir(111): a) Spectra of graphene on Ir(111) (blue) and a monolayer HAT/gr/Ir(111) (orange) before subtraction, b) difference spectrum (purple). The difference spectrum is calculated by subtracting the monolayer HAT and the graphene/Ir(111) spectra from a). Shown as reference in b) is the spectrum of multilayer HAT (not subtracted)

Table S1. Positions of the HOMO levels of HAT on Ag(111) and graphene/Ir(111) from the UPS spectra. Binding energy is given in eV for monolayer/multilayer coverages of HAT. Due to overlap with the Ag 4*d* bands, some HOMO levels cannot be identified in the monolayer spectrum.

Ag(111)	2.2/2.6	3.3/3.6	5.2/5.6	-/6.7	-/8.2
Gr/ Ir(111)	1.5/1.8	2.8/3.1	4.8/5.0	6.5/6.7	7.4/7.6

Additional information on the x-ray photoelectron spectroscopy (XPS) data for HAT/graphene/Ir(111)

The ratio C(graphene):C(HAT) used in fitting the data was determined from the exponential attenuation by the top HAT layers for both samples, using

$$I_{HAT} = I_{HAT}^{0} \cdot \left(1 - exp\left(-\frac{d_{HAT}}{\lambda}\right)\right)$$

$$I_{Gr} = I_{Gr}^{0} \cdot \left(1 - exp\left(-\frac{d_{Gr}}{\lambda}\right)\right) \cdot exp\left(-\frac{d_{HAT}}{\lambda}\right)$$

$$\frac{I_{HAT}}{I_{Gr}} = \frac{N_{HAT}}{N_{Gr} \cdot exp\left(-\frac{d_{HAT}}{\lambda}\right)}$$

where N_{HAT} and N_{Gr} are the carbon density in the HAT and graphene layer (from literature and from this manuscript, respectively). d_{HAT} is the thickness of the HAT layer(s) as determined by the quartz microbalance (QMB), calibrated to one monolayer by STM.. λ is the inelastic mean free path (IMFP), which we calculated from the NIST Inelastic Mean Free Path database[4] (using the TPP-2M equation[5]). For C1s photoelectrons excited with Mg K_{α} X-rays (kinetic energy 963 eV) this gives an IMFP of 2.8 nm.

Using the atomic sensitivity factors (ASF) from the Handbook of X-ray Photoelectron Spectroscopy[6] we compare the O1s and C1s HAT core levels in the table below. The second column gives the fitted peak area, the third the ASF, the fourth the peak area corrected for the ASF. In the fifth column, these areas are divided for comparison to the HAT stoichiometry in the sixth column (which is 12:12:6, see Scheme 1 in the manuscript).

Table S2: Stoichiometry calculations for monolayer HAT/graphene/Ir(111)

**Peak**	**Area (counts · eV)**	**A.S.F.**	**Corrected area**	**Corrected ratio**	**Atomic ratio**
**HAT C1**	4985.75	0.296	16843.75	2.099121196	2
**HAT C2**	4985.75	0.296	16843.75	2.099121196	2
**HAT O**	5705.2	0.711	8024.19128	1	1

The stoichiometry calculated from the XPS data agrees within 5% with the expected stoichiometry.

References

[1] A.T. N'Diaye, J. Coraux, T.N. Plasa, C. Busse, T. Michely, Structure of epitaxial graphene on Ir(111), *New J. Phys.,* **2008**, 10(4), 043033, https://doi.org/10.1088/1367-2630/10/4/043033

[2] S. Hamäläinen, M. Stepanova, R. Drost, P. Liljeroth, J. Lahtinen, J. Sainio, Self-Assembly of Cobalt-Phthalocyanine Molecules on Epitaxial Graphene on Ir(111), *J. Phys. Chem. C*, **2012**, 116, 38, 20433-20437, https://doi.org/10.1021/jp306439h

[3] F.S. Khokhar, R. van Gastel, B. Poelsema, Role of topographical defects in organic film growth of 4,4′-biphenyldicarboxylic acid on graphene: A low-energy electron microscopy study, *Phys. Rev. B*, **2010**, 82(20), 205409, https://doi.org/10.1103/PhysRevB.82.205409

[4] C.J. Powell, A. Jablonski, NIST Electron Inelastic-Mean-Free-Path Database, Version 1.2. National Institute of Standards and Technology, Gaithersburg, MD, **2010**, http://dx.doi.org/10.18434/T48C78

[4] S. Tanuma, C.J. Powell, D.R. Penn, Calculations of electron inelastic mean free paths. V. Data for 14 organic compounds over the 50–2000 eV range. *Surf. Interface Anal.*, **1994**, 21(3), 165, https://doi.org/10.1002/sia.740210302

[5] J.F. Moulder, W.F. Stickle, P.E. Sobol, K.D. Bomben, Handbook of X-ray Photoelectron Spectroscopy, Perklin Elmer Corporation, **1992**
